# Alteration of White Matter in Patients with Central Post-Stroke Pain

**DOI:** 10.3390/jpm11050417

**Published:** 2021-05-15

**Authors:** Jung Geun Park, Bo Young Hong, Hae-Yeon Park, Yeun Jie Yoo, Mi-Jeong Yoon, Joon-Sung Kim, Seong Hoon Lim

**Affiliations:** 1 Department of Rehabilitation Medicine, St. Vincent’s Hospital, College of Medicine, The Catholic University of Korea, 93 Jungbu-daero, Paldal-gu, Suwon 16247, Korea; jgp0123@naver.com (J.G.P.); byhong@catholic.ac.kr (B.Y.H.); nugry@naver.com (Y.J.Y.); allogen@naver.com (M.-J.Y.); svpmr@chol.com (J.-S.K.); 2 Department of Rehabilitation Medicine, Seoul St. Mary’s Hospital, College of Medicine, The Catholic University of Korea, Seoul 06591, Korea; hy2park@naver.com

**Keywords:** central post-stroke pain, stroke, thalamic pain, spinothalamic tract, anterior thalamic radiation, superior thalamic radiation, white mater, diffusion tensor imaging, DTI

## Abstract

A stroke may be followed by central post-stroke pain (CPSP), which is characterized by chronic neuropathic pain. The exact mechanism has not yet been fully uncovered. We investigated alterations in the white matters in patients with CPSP, compared with stroke patients without CPSP and normal controls. Our retrospective cross-sectional, case-control study participants were assigned to three groups: CPSP (stroke patients with CPSP (*n* = 17)); stroke control (stroke patients without CPSP (*n* = 26)); and normal control (normal subjects (*n* = 34)). The investigation of white matter for CPSP was focused on the values of fiber numbers (FN) and fractional anisotrophy (FA) for spinothalamic tract (STT), anterior thalamic radiation (ATR), superior thalamic radiation (STR) and posterior thalamic radiation (PTR), and corticospinal tract (CST) was measured. The FA for the STT and STR of the CPSP group were lower than those for the stroke control and normal control groups. The FA of CST and ATR did not differ between the CPSP and stroke groups, but both differed from the normal control. The FA of PTR in the stroke control group differed from the normal control group, but not from the CPSP group. The FN of CST, STT, ATR, and STR for the CPSP and stroke control groups did not differ from each other, but both differed from those of normal controls. FN of PTR did not differ between the CPSP and normal control groups. The alterations in the spinothalamic tract and superior thalamic radiation after stroke would play a role in the pathogenesis of CPSP.

## 1. Introduction

Central post-stroke pain (CPSP) can occur after a cerebrovascular accident and is characterized by neuropathic pain syndrome [1]. It has characteristics of stimulation-independent pain, such as shooting, burning, or electric shock-like sensations and paresthesia [1,2,3]. The prevalence of CPSP is about 8% in all stroke patients but is as high as 18% among sensory deficit stroke patients [1]. Thermal or pinprick hypersensitivity is more common in CPSP than in non-CPSP patients, which indicates that spinothalamic tract hyperexcitability might be an underlying mechanism of CPSP [4].

Several theories suggest that brain regions such as the somatosensory cortex, inferior parietal lobe, cingulate gyrus, lateral thalamus, and medial meniscus are responsible for the pathophysiologic mechanism of CPSP [5,6,7,8]. Theories about the pathogenesis of CPSP include disinhibition, central sensitization, alterations in spinothalamic tract function, and thalamic changes [5,6,9,10,11]. Disinhibition theory suggests that a lesion in the lateral thalamus causes disinhibition of the medial spinothalamic tract pain signaling pathway, which ultimately causes CPSP [12]. The explanation in terms of central sensitization suggests that anatomical, neurochemical, and inflammatory changes in Central nervous system(CNS) lesions trigger neuronal excitability, which generates central sensitization and chronic pain [13]. Investigations such as this into the pathogenesis of CPSP are underway, but the pathophysiologic mechanism of CPSP has yet to be fully explained. Recently, several reports have demonstrated that structural changes in the spinothalamic tract might be associated with CPSP development [14,15,16]. In addition, hypometabolism of the ipsilesional primary motor cortex has been reported in patients with CPSP [17].

Thus, we hypothesized that central pain after stroke may be based on morphological and structural changes in the white matter of the thalamus, primary sensory cortex, and primary motor cortex. We investigated alterations in the spinothalamic tract (STT) and thalamocortical tract; anterior thalamic radiation (ATR), superior thalamic radiation (STR), and posterior thalamic radiation (PTR); and the corticospinal tract (CST) in three groups: stroke patients with CPSP, stroke patients without CPSP, and normal controls.

## 2. Materials and Methods

### 2.1. Study Design and Participants

This was a retrospective case—control study. Subjects were assigned to three groups (Table 1): CPSP (stroke patients with CPSP (*n* = 17)); stroke control (stroke patients without CPSP (*n* = 26)); and normal controls (*n* = 34). The CPSP and stroke control groups comprised 43 patients with first-ever supratentorial unilateral stroke involving the thalamus or basal ganglia, and all met the following criteria: (1) diagnosed with first-ever supratentorial unilateral stroke involving the thalamus or basal ganglia and (2) 3.0-T magnetic resonance imaging (MRI) scan and brain diffusion tensor imaging (DTI) performed 6 months after onset. Exclusion criteria were: (1) recurrent stroke; (2) diagnosis of brain complications after stroke, such as hydrocephalus; (3) underlying degenerative brain disease, such as Parkinson’s disease; and (4) other pain syndrome, such as complex regional pain syndrome, fibromyalgia, or other rheumatologic disease. Normal controls were age- and sex-matched subjects chosen from the data from the Health Promotion Center of our institution [18].

CPSP on the hemiplegic side was defined using as a score of 4 or higher on a 10-point visual analog scale (VAS), completed at 6 months after onset. The stroke control group included patients without central post-stroke pain at 6 months after onset. Visual analog scales (VAS) were used for evaluations during outpatient visits; patients were instructed to rate their pain from zero to 10, where zero represented no pain and 10 was the worst pain imaginable. Patients with central pain of ≤3 on the VAS were excluded from the study to avoid ambiguity in group assignments. Potential normal controls were excluded for: (1) a history of neurological disorder determined through medical examination and (2) structural abnormalities on their scan. Considering the process of neurologic recovery, 6 months after stroke onset was defined as the completion of neurological recovery [19].

The present study was an observational investigation of CPSP, so the exact sample size was not calculated beforehand. However, the sample sizes of previous studies varied from 2 to 14 [16,20,21]; thus, it was determined that the sample size for this study would be more than 14 subjects. Moreover, to increase the accuracy of our results, we decided to include a stroke control group 1.5 times the size of the CPSP group and a normal control group twice the size of the CPSP group.

The study protocol was reviewed and approved by the Institutional Review Board of Catholic University, College of Medicine (Registry No. VC20RASI0185); the requirement for informed consent was waived by the board.

### 2.2. Diffusion Tensor Imaging Acquisition and Image Processing

Diffusion tensor imaging was performed using a 3.0-T magnetic resonance imager (MAGNETOM^®^ Verio, Siemens, Erlangen, Germany) equipped with a six-channel head coil. Data were acquired in the form of single-shot spin-echo echo-planar images, with axial slices covering the whole brain across 76 interleaved slices of 2.0 mm thickness (no gap; repetition time/echo time = 14,300/84 ms; field of view = 224 × 224 mm^2^; matrix 224 × 224; voxel size 1 × 1 × 2 mm^3^ (isotropic); number of excitations = 1). Diffusion sensitizing gradients were applied in 64 noncollinear directions with a b-value of 1000 ms/mm^2^. The b = 0 images were scanned before acquisition of the diffusion-weighted images, with 65 volumes in total [22,23].

Fiber tracking was based on the fiber assignment continuous tracking (FACT) algorithm and a multiple regions of interest (ROIs) approach using DTI-studio [24,25]. The termination criteria were fractional anisotropy (FA) < 0.2 and an angle change > 60 degrees [25].

### 2.3. Diffusion Tensor Tractography

**Figure 1:**
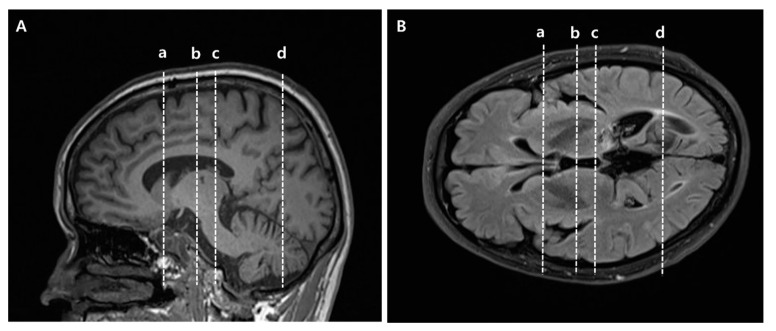
Specific anatomical locations of seed and target regions of interest (ROIs) of anterior thalamic radiation (ATR) and posterior thalamic radiation (PTR) in slice levels in brain MR images. (**A**) Sagittal view for ROIs, (**B**) Axial view for ROIs. (a) Target ROI of ATR where the frontal and temporal lobes are separated. Equivalent slice level to (a) in Supplementary Materials Figure S3. (b) Seed ROI of ATR located on the anterior part of the thalamus where the substantia nigra first appears. Equivalent slice level to (b) in Supplementary Materials Figure S3. (c) Seed ROI of PTR located on the posterior part of the thalamus, where the posterior tip of the putamen lies. Equivalent slice level to (c) in Supplementary Materials Figure S4. (d) Target ROI of PTR located below the parieto-occipital sulcus. Equivalent slice level to (d) in Supplementary Materials Figure S4.

To reconstruct the CST, the seed ROI was placed on the mid-pons portion of the CST in the axial plane, and the target ROI was the primary motor cortex (Supplementary Materials Figure S1) [22]. STT was reconstructed using two ROIs, with the seed ROI placed on the posterolateral medulla (posterior to the inferior olivary nucleus, anterior to the inferior cerebellar peduncle, and lateral to the medial lemniscus) in the axial plane, and the target ROI was the primary somatosensory cortex (Supplementary Materials Figure S2) [26,27]. For reconstruction of the ATR, the seed ROI was located on the anterior part of the thalamus in the coronal plane where the substantia nigra first appears, and the target ROI was at the slice level where the frontal and temporal lobes were separated in the coronal plane (Supplementary Materials Figure S3) [28]. PTR was reconstructed using two ROIs, with the seed ROI placed on the posterior thalamus at the slice level where the posterior tip of the putamen lies in the coronal plane, and the target ROI was the occipital lobe below the parieto-occipital sulcus (Supplementary Materials Figure S4). To reconstruct STR, the seed ROI was on the middle thalamus in the coronal plane at the midpoint slice level between the ATR and PTR seed ROI, and the target ROI was the entire ipsilateral hemisphere within a transverse section above the corpus callosum in the axial plane (Supplementary Materials Figure S5) [29]. As the seed and target ROIs of ATR and PTR were complicated, specific anatomical locations are provided in Figure 1. The FA values were measured in the seed ROIs of each tract in both hemispheres, and FN values were measured in both hemispheres of all patients. The normalization ratios for FN and FA values were calculated using the following formula in the CPSP and non-CPSP groups: data of affected side/data of non-affected side [25]. For the control group, the normalization ratio for FN and FA values was calculated as left/right [22].

### 2.4. Statistical Analysis

The FN and FA values in the three groups are presented as the median (interquartile range: first–third quartiles). The Kruskal–Wallis test was conducted to evaluate differences among the three groups, followed by the Mann–Whitney U-test with the Bonferroni correction. The Mann–Whitney U-test and the Bonferroni correction were two-tailed, and *p*-values ≤ 0.0166 were deemed significant. All statistical analyses were performed using SPSS software for Windows (ver. 21.0; SPSS, Inc., Chicago, IL, USA).

## 3. Results

Demographic and clinical characteristics of the three groups are presented in Table 1. The distributions of stroke type, brain injury location, and comorbidity did not differ among the three groups. The FN and FA values of the CST, STT, ATR, STR, and PTR for all three groups are presented in Table 2.

**Figure 2:**
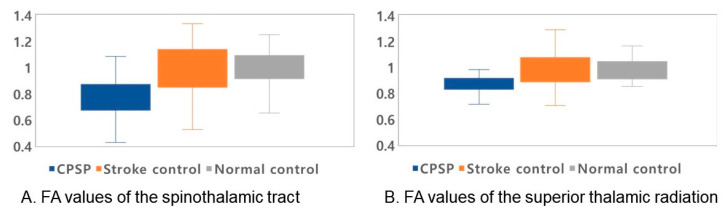
Normalized fractional anisotropy (FA) values of the spinothalamic tract (STT) and superior thalamic radiation (STR). The median values with quartiles are shown as lines. For all values, the rectangular shape shows the range between the first and third quartiles. (**A**) FA values for STT for all groups. The FA value of STT in the CPSP group was lower than those in the stroke control and normal control groups (*p*-value < 0.001). (**B**) FA values for STR for all groups. The FA value of STR in the CPSP group was lower than those in the stroke control and normal control groups (*p*-values 0.03, and 0.01, respectively).

**Figure 3:**
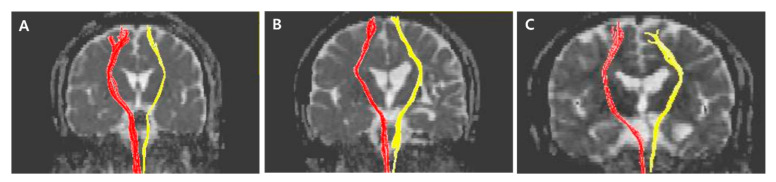
Representative diffusion tensor tractography images of the spinothalamic tract in typical subjects from the (**A**) CPSP, (**B**) stroke control, and (**C**) normal control groups. The non-affected tract is shown in red, and the affected tract in yellow.

**Figure 4:**
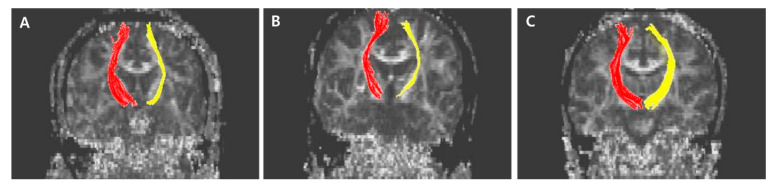
Representative diffusion tensor tractography images of superior thalamic radiation in typical subjects from the (**A**) CPSP group, (**B**) stroke control, and (**C**) normal control groups. The non-affected tract is shown in red, and the affected tract in yellow.

The normalized FA values of STT in the CPSP group were lower than those in the stroke control group and normal control groups; 0.79 for CPSP, 0.98 for non-CPSP, 0.98 for control (Figure 2A). The normalized FA values of STR in the CPSP group were lower than those in the stroke control group and normal control groups; 0.88 for CPSP, 1.00 for non-CPSP, 0.97 for control (Figure 2B). There were no differences in the FA values of STT and STR between the stroke control and normal control groups. The normalized FN values of STT and STR were lower in the CPSP and stroke control group than in the normal control group. However, there was no significant difference between the CPSP and stroke control for the normalized FN of STT and STR. Representative DTIs of STT in all three groups are shown in Figure 3, and those of STR in Figure 4.

The normalized FA values of CST were lower in the CPSP and stroke control group than in the normal control group. No significant difference was shown between CPSP and stroke control for normalized FA values of CST. There was no significant difference among all three groups for normalized FA values of ATR. The FN values of CST and ATR were lower in the CPSP and stroke control group than in the normal control group, and there was no difference between CPSP and stroke control. The normalized FA and FN value of PTR among three groups were not different from each other.

## 4. Discussion

In conceptualizing this study, we considered that structural changes in several white matters involved in the sensory pathway, i.e., STT, ATR, STR, and PTR, would be related to the pathogenesis of CPSP. Consistent with our results, a recent study demonstrated that partial injury of the STT in patients with intracranial hemorrhage may be related to the pathogenesis of CPSP [14]. The ventral posterolateral nucleus of the thalamus is closely related to CPSP, as the superior thalamic radiation projects fibers from the ventral nucleus group of the thalamus to the precentral and postcentral gyrus [30]. Our results show that the FA values of STT and STR in the CPSP group were lower than those in the stroke control and normal control groups. Our results are consistent with the anatomical function of the thalamus in finding that neural injury of STR in the thalamocortical pathway is associated with the pathophysiologic mechanism of CPSP.

Considering a previous study that found hypometabolism of the primary motor cortex in CPSP [17], we also investigated changes in the CST in relation to CPSP. However, the CST values in the CPSP group did not differ from those in the stroke control group, and the CST values of the CPSP and stroke control groups were lower than those of the normal controls, as expected. Thus, we suggest that changes in STT and STR may be related to CPSP. Despite metabolic changes in the motor cortex, the white matter was not altered by the development of CPSP.

Our study had several limitations, such as a relatively small sample size, the subjective nature of pain, and the cross-sectional design itself. Thus, we used several methods to overcome bias. First, for overcoming the relatively small sample size, we undertook the analyses by comparing three groups: CPSP, stroke control, and normal control. In addition, we enrolled the larger numbers of sample size compared to previous studies [16,20,21]. Thus, we drew out the alterations of white matter for CPSP. Second, for reducing bias relevant to subjective nature of pain itself, we excluded patients with ambiguous pain levels, as measured by VAS scores between one and three. However, the participants’ pain, especially neuropathic pain, is necessarily measured subjectively using a self-reported measure, making it difficult to present it an objective or quantified manner. In addition, sensory abnormalities in stroke patients could be expressed as abnormal sensations of pain or as thermal, touch, or vibration abnormalities. Various types of sensations should be considered in further studies to clarify the specific mechanism of CPSP. Finally, our study was a cross-sectional study and, therefore, did not reveal longitudinal changes in white matter. Further research is needed to address the remaining questions regarding CPSP.

In conclusion, changes in STT and STR may play a role in CPSP among patients with chronic supratentorial stroke.

## Figures and Tables

**Table 1:** Participants’ demographic data.

	CPSP	Stroke Control	Normal Control	*p*
Subjects, *n*	17	26	34	
Age, years	57.3 (51.75–62.25)	53.17 (44.0–67.25)	57.9 (51.0–64.0)	
Female sex, *n* (%)	4 (23.5)	8 (30.8)	10 (29.4)	0.867
Stroke type, *n* (%)				
Hemorrhage	9 (52.9)	14 (53.8)	N/A	0.954
Infarction	8 (47.1)	12 (46.2)	N/A	
Brain injury location, *n* (%)				
Basal ganglia	10 (58.8)	15 (57.7)	N/A	0.810
Thalamus	6 (35.3)	8 (30.8)	N/A	
Both	1 (5.9)	3 (11.5)	N/A	
Stroke side, Left/Right, *n* (%)	12/5 (70.6/29.4)	18/8 (69.2/30.8)	N/A	0.925
Comorbidity				
Diabetus Mellitus	4 (23.5)	1 (3.8)	5(14.7)	0.159
Hypertension	9 (52.9)	16 (61.5)	12 (35.3)	0.118
Arterial fibrillation	0 (0)	2 (7.7)	0 (0)	0.133

Values are the median (interquartile range: first–third quartiles), or number (*n*) (%). *p*-values were tested using Pearson’s chi-square test, CPSP; central post-stroke pain, N/A; not applicable.

**Table 2:** FN, FA values of CST, STT, ATR, STR, PTR by group.

	Values	CPSP	P1	Stroke Control	P2	Normal Control	P3
CST	FN	0.31 (0.06–0.76)	0.881	0.33 (0.05–0.71)	0.003	0.86 (0.46–1.14)	0.001
	FA	0.75 (0.65–0.94)	0.881	0.79 (0.73–0.90)	<0.001	0.96 (0.91–1.03)	<0.001
STT	FN	0.47 (0.31–0.69)	0.728	0.40 (0.09–0.86)	<0.001	1.4 (0.59–2.73)	<0.001
	FA	0.79 (0.68–0.85) *	<0.001	0.98 (0.85–1.11) *	0.994	0.98 (0.91–1.10) *	<0.001
ATR	FN	0.76 (0.65–0.94)	0.823	0.68 (0.60–1.10)	0.005	1.00 (0.90–1.26)	0.002
	FA	0.96 (0.83–1.09)	0.593	0.99 (0.85–1.09)	0.551	1.01 (0.95–1.06)	0.337
STR	FN	0.45 (0.28–0.62)	0.785	0.34 (0.17–0.70)	<0.001	0.91 (0.67–1.40)	<0.001
	FA	0.88 (0.83–0.93) *	0.004	1.00 (0.90–1.07) *	0.836	0.97 (0.91–1.04) *	0.001
PTR	FN	0.32 (0.18–0.86)	0.172	0.60 (0.40–1.22)	0.748	0.69 (0.25–1.27)	0.223
	FA	0.93 (0.87–1.00)	0.087	1.03 (0.93–1.12)	0.005	0.91 (0.84–0.98)	0.734

Values are the median (interquartile range: first–third quartiles) and normalized. Normalized values of FA, FN in CPSP and non-CPSP group are shown as affected/non-affected. For normal control, normalized values are shown as left/right. CST, corticospinal tract; STT, spinothalamic tract; ATR, anterior thalamic radiation; STR, superior thalamic radiation; PTR, posterior thalamic radiation; FA, fractional anisotropy; FN, fiber number. * *p* < 0.05, three groups were compared using the Kruskal–Wallis test. P1; comparison between CPSP and non-CPSP groups with the Mann–Whitney U-test with the Bonferroni correction (*p* ≤ 0.0166 deemed to be significant). P2; comparison between non-CPSP and control groups with the Mann–Whitney U-test with the Bonferroni correction (*p* ≤ 0.0166 deemed to be significant). P3; comparison between control and CPSP groups with the Mann–Whitney U-test with the Bonferroni correction (*p* ≤ 0.0166 deemed to be significant).

## Data Availability

The data presented in this study are available on request from the corresponding author.

## Supplementary Materials

The following are available online at https://www.mdpi.com/article/10.3390/jpm11050417/s1, Figure S1: (ROIs) used to reconstruct the corticospinal tract (CST) on DTI and representative DTT images of the corticospinal tract; Figure S2: Regions of interest (ROIs) used to reconstruct the spinothalamic tract (STT) on DTI and representative DTT images of the spinothalamic tract; Figure S3: Regions of interest (ROIs) used to reconstruct anterior thalamic radiation (ATR) on DTI and representative DTT images of anterior thalamic radiation; Figure S4: Regions of interest (ROIs) used to reconstruct the posterior thalamic radiation (PTR) on DTI and representative DTT images of posterior thalamic radiation; Figure S5: Regions of interest (ROIs) used to reconstruct the superior thalamic radiation (STR) on DTI and representative DTT images of superior thalamic radiation.
