# Supplementary material for: Alteration of White Matter in Patients with Central Post-Stroke Pain
Source: J Pers Med. 2021 May 15;11(5):417. doi: 10.3390/jpm11050417 (PMC8156708; doi:10.3390/jpm11050417)

Supplementary Figure 1. Regions of interest (ROIs) used to reconstruct the corticospinal tract (CST) on DTI and representative DTT images of the corticospinal tract. (A) The solid line indicates the seed ROI of CST at mid-pons in an axial color map. (B) The grey area surrounded by white stippled lines indicates the target ROI of CST in an axial brain MRI. (C) Stippled line in the sagittal color map at mid-pons level. (D) Representative DTT image of the CST in a typical subject in the normal control group.

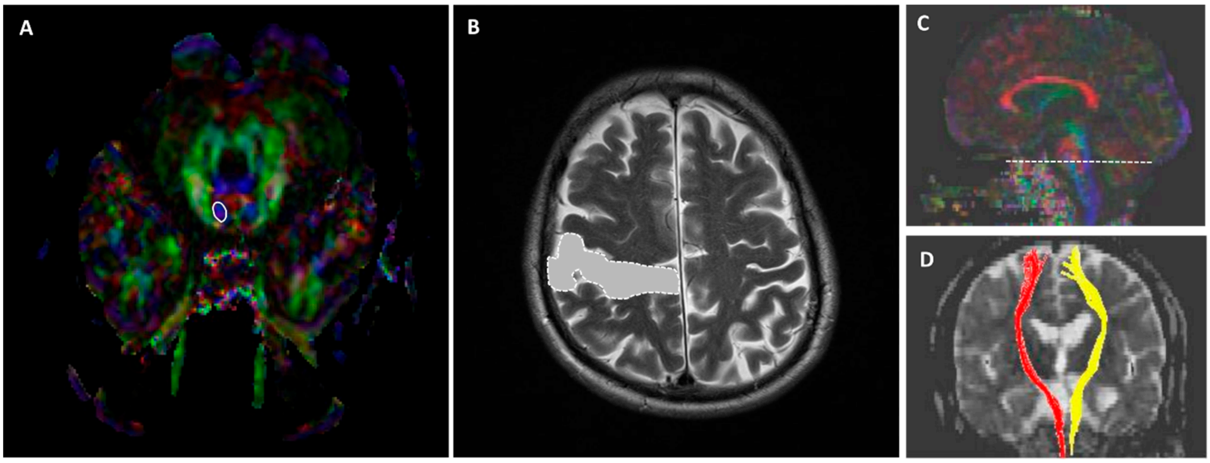

Supplementary Figure 2. Regions of interest (ROIs) used to reconstruct the spinothalamic tract (STT) on DTI and representative DTT images of the spinothalamic tract. (A) The solid line indicates the seed ROI of STT on the posterolateral medulla in the axial color map. (B) The grey area surrounded by white stippled lines indicates the target ROI of STT in an axial brain MRI. (C) Stippled line in the sagittal color map at medulla level. (D) Representative DTT images of STT in a typical subject in the normal control group.

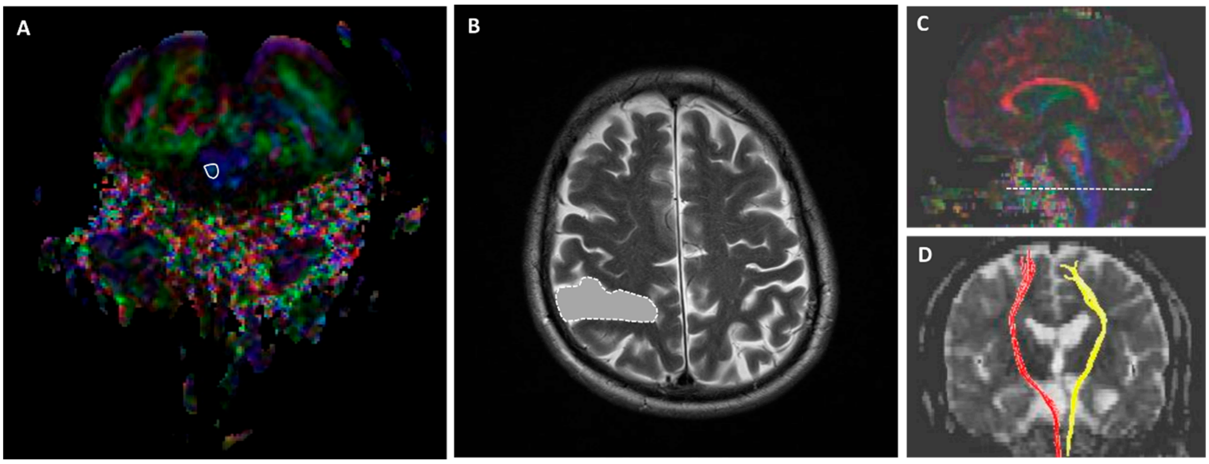

Supplementary Figure 3. Regions of interest (ROIs) used to reconstruct anterior thalamic radiation (ATR) on DTI and representative DTT images of anterior thalamic radiation. (A) The solid line indicates the seed ROI of ATR on the anterior part of the thalamus in a coronal view. (B) The stippled line indicates the target ROI of ATR in the coronal view. (C) (a) indicates the slice level of the target ROI, and (b) indicates the slice level of the seed ROI in the sagittal plane. (D) Representative DDT image of ATR in a typical subject in the normal control group.

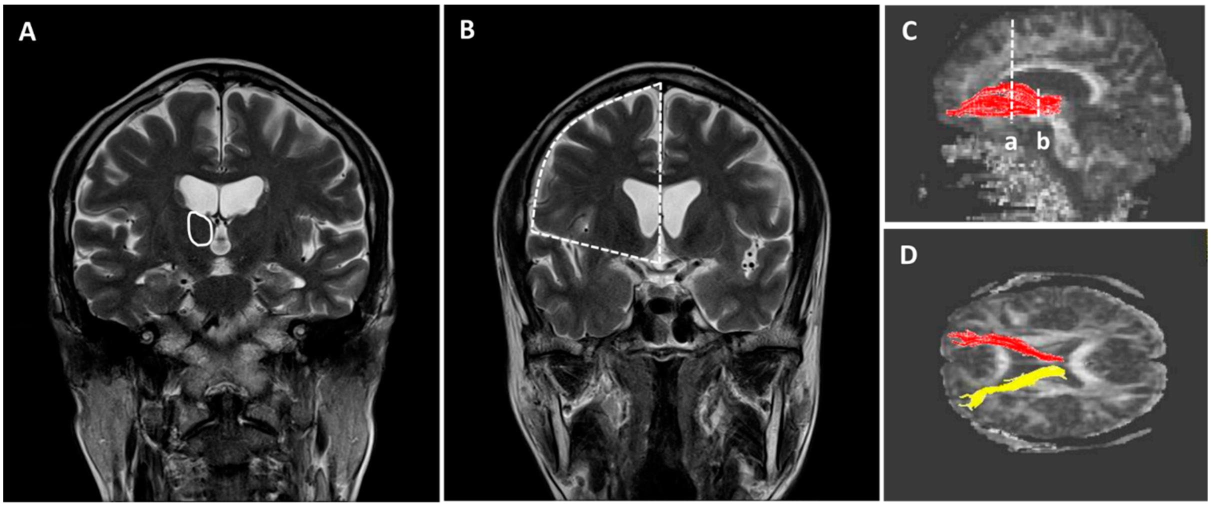

Supplementary Figure 4. Regions of interest (ROIs) used to reconstruct the posterior thalamic radiation (PTR) on DTI and representative DTT images of posterior thalamic radiation. (A) The solid line indicates the seed ROI of PTR on the posterior thalamus in a coronal view. (B) The stippled line indicates the target ROI of PTR in a coronal view. (C) (c) indicates the slice level of the seed ROI, and (d) indicates the slice level of the target ROI in sagittal plane. (D) Representative DTT image of PTR in a typical subject in the normal control group.

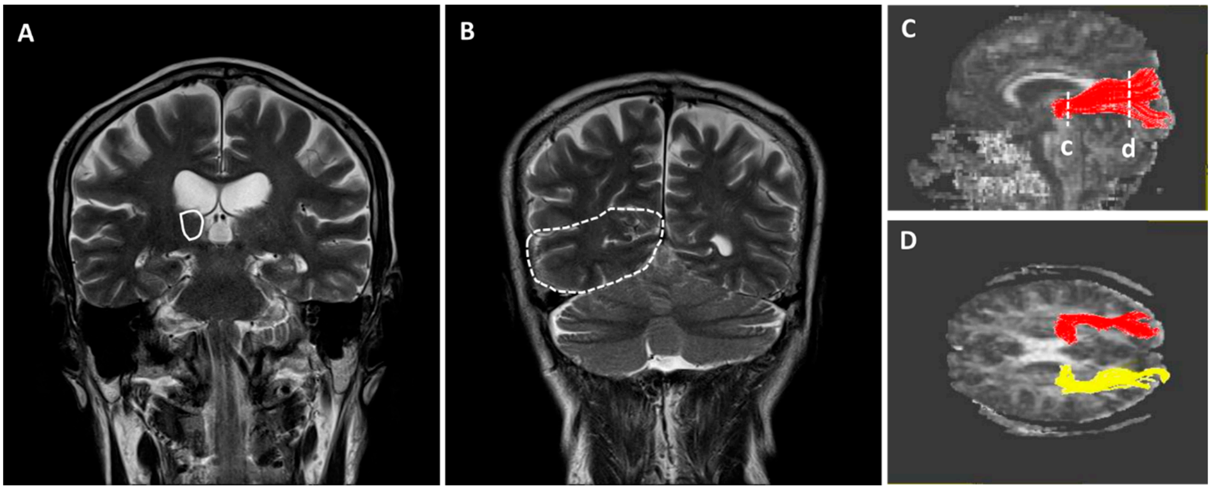

Supplementary Figure 5. Regions of interest (ROIs) used to reconstruct the superior thalamic radiation (STR) on DTI and representative DTT images of superior thalamic radiation. (A) The solid line indicates the seed ROI of STR on the middle part of the thalamus in a coronal view. (B) The stippled line indicates the target ROI of STR in the axial view. (C) (e) indicates the axial level of the seed ROI, and (f) indicates the axial level of the target ROI. (D) Representative DTT image of STR in a typical subject in the control group.

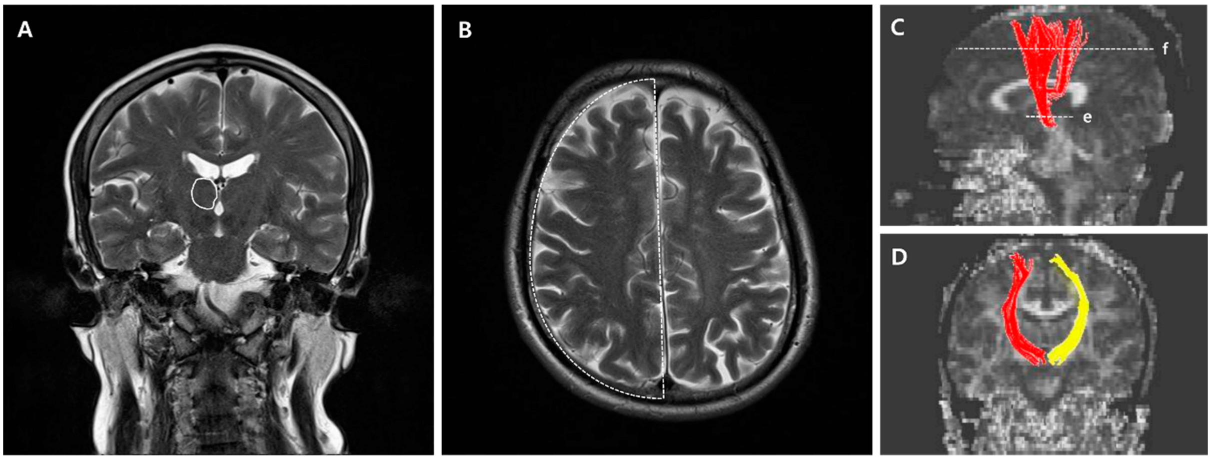

Supplement: Supplementary file 1 [file jpm-11-00417-s001.zip › jpm-1203963-supplementary.pdf]
